# Acute heat stress brings down milk secretion in dairy cows by up-regulating the activity of the milk-borne negative feedback regulatory system

**DOI:** 10.1186/1472-6793-9-13

**Published:** 2009-06-29

**Authors:** Nissim Silanikove, Fira Shapiro, Dima Shinder

**Affiliations:** 1 Biology of Lactation Laboratory, Inst. of Animal Sciences, Agricultural Research Organization, PO Box 6, Bet Dagan 50250, Israel

## Abstract

**Background:**

The objective of this study was to determine if acute heat stress (HS) decreases milk secretion by activating the milk-borne negative feedback system, as an emergency physiological response to prevent a life-threatening situation. To induce HS, summer acclimatized dairy cows were exposed to full sun under mid-summer Mediterranean conditions, with and without conventional cooling procedures.

**Results:**

Exposure to HS induced a rapid and acute (within 24 h) reduction in milk yield in proportion to the heat load. This decrease was moderated by cooler night-time ambient temperature. The reduction in milk yield was associated with corresponding responses in plasminogen activator/plasminogen-plasmin activities, and with increased activity (concentration) of the (1–28) N-terminal fragment peptide that is released by plasmin from β-casein (β-CN (1–28)). These metabolites constitute the regulatory negative feedback system. Previously, it has been shown that β-CN (1–28) down-regulated milk secretion by blocking potassium channels on the apical aspects of the mammary epithelial cells.

**Conclusion:**

Here we demonstrate that the potassium channels in mammary tissue became more susceptible to β-CN (1–28) activity under HS. Thus, the present study highlighted two previously unreported features of this regulatory system: (i) that it modulates rapidly in response to stressor impact variations; and (ii) that the regulations of the mammary epithelial potassium channel sensitivity to the inhibitory effect of β-CN (1–28) is part of the regulatory system.

## Background

In hot climates, high ambient temperatures, and high direct and indirect solar radiation, wind speed and humidity, are the main environmental stressing factors that impose stress on animals [1,2]. Cattle have a higher metabolic rate than most other domestic ruminants, and a poorly developed water retention mechanism in the kidney and gut [1,3]. Furthermore, as a consequence of aggressive selection for milk production over the last 5 decades, modern cows in Israel and the USA produce 40–70 L of milk per day, compared with 10 L day^-1 ^or less in their ancestors. Each 10 L day^-1 ^of milk yield roughly doubles the metabolizable energy requirement of cows, and ~35% of this energy is dissipated as heat [2]. High-yielding cows are affected more than low-yielding ones [4,5] because the upper critical temperature shifts downward as milk production, feed intake, and heat production increase [1].

Acclimation involves phenotypic responses to environmental changes, which are reflected in hormonal signals and also in alterations in target tissue responsiveness to hormonal stimuli [6,7]. The time required for acclimation varies according to tissue types, and ranges from a few days to several weeks; for example, changes in metabolism in response to HS occur over a few days [8,9].

However, this picture does not cover all situations; some external environmental stresses, such as dehydration [10,11] and acute HS [1,12,13], may very quickly (within 24 to 48 h) take animals beyond their current acclimatized-adaptive range, which necessitates the induction of emergency physiological responses in order to avoid sudden death. Such immediate measures include an acute reduction in milk yield, because milk production, particularly in high-yielding dairy cows, intensifies the effects of these external stresses [1,10].

Milk secretion and mammary function are regulated acutely by local autocrine feedback mechanisms that involve milk-borne factors which are sensitive to the frequency and efficiency of milking [14,15]. Sustained changes in the frequency of milking and milk secretion are associated metabolic adaptations [16] and with longer-term adaptations in the degree of differentiation and, ultimately, the number of mammary epithelial cells [17,18]. Consequently, such changes may affect the above-described homeostatic processes. It has been hypothesized that the fast modulation of milk secretion in response to external factors, such as emotional stress, and harsh physical conditions such as heat stress and water deprivation also depends on such a negative-feedback regulatory system, which increases the potential for survival in response to stress [19,20]. This negative feedback system was shown to comprise an endogenous milk enzymatic system, the plasminogen activator (PA)-plasminogen (PG)-plasmin (PL), that specifically forms a β-casein (CN) fragment (f) (1–28) from β-CN, which acts as the negative control signal by closing potassium channels on the apical membrane of the epithelial cells of the mammary gland [19,20]. Down-regulation of these channels induces undefined inwardly directed cellular signals that inhibit milk secretion. Interestingly, a further activation of the PA-PG-PL system, which was coupled with more extensive degradation of casein induced involution of the mammary gland in lactating goats and cows and forcefully activated the innate immune system [20-23]. Based on these findings, a casein hydrolyzate preparation was developed to reduce the suffering from mammary gland engorgement associated with abrupt cessation of milking (the conventional procedure to induce involution in modern dairy cows) [24] and to treat and prevent common clinical and subclinical infections of the udder in dairy cows [25,26].

The concept that PA-PG-PL-β-CN f(1–28) is involved in milk-born negative feedback regulation of milk secretion was supported experimentally under conditions that simulated stress (intramammary treatment with dexamethasone) [19,27], and by exposure of cows to dehydration [19].

The aims of the present study were:

**1**. to examine the hypothesis that the PA-PG-Pl-β-CN f (1–28) system is involved in regulation of milk secretion under acute HS;

**2**. to assess whether this system is sufficiently sensitive to react to diurnal changes in environmental HS;

**3**. to examine whether the potassium channels that act in response to β-CN f (1–28) are also regulated.

## Methods

### Ethics

All protocols were approved by the Institutional Animal Care Committee of the Agricultural Research Organization, which is the legitimate body for such authorizations in Israel.

### Study layout

The experiment was carried out at the height of summer (late July) in the experimental dairy herd of the Agricultural Research Organization, at the Volcani Center at Bet Dagan. Eighteen Israeli Holstein cows, between their second and fourth lactations, with milk yields of 48–52 l day^-1 ^were subjected to the study. The cows were allocated to three treatments, each of six cows, according to lactation number and milk yield (MY). The treatments were: treatment C – the cows had access to shade and benefited from the conventional cooling procedures in the herd; treatment D – the cows were denied access to shade, but benefited from the cooling procedures; and treatment E – the cows were denied access to both shade and cooling procedures. The cows were held in small yards; those in treatment C had 10 m^2 ^of a shaded slatted floor and 10 m^2 ^of an unshaded concrete – surfaced yard per animal, whereas those in treatments D and E had 20 m^2 ^of an unshaded concrete – surfaced yard per animal. The cows were fed before and during the experiment with a typical Israeli total mixed ration (17% protein) containing 65% concentrate and 35% forage, which was offered *ad lib *in managers; water was available at all times. At the noon milking before the start of the experiment, milk samples were taken from all cows, and then the cows were allocated to treatments. Milk samples were taken during the morning and noon milking of the subsequent two days; after the last measurement of rectal temperature at 1400 on the second day the cows were returned to their original group.

The cows were milk three times daily (0530, 1230, and 2130), and the and exact milking times were individually recorded automatically [13]. The cooling system has been described previously [28]. Briefly, an array of fans produced air velocities of 2 m/second or more from 0600 to 2400. In addition, fans and sprinklers were sequentially activated to repeat cycles of wetting (0.5 min) and ventilation (4.5 min) for seven 0.5-h periods every 1.5 to 2 h, between 0730 and 1830.

### Ambient conditions

Air temperature and relative humidity were recorded at a meteorological station located 1.5 km from the farm. Average maximal (noon) temperature during the three days of the experiments were 31, 35 and 37°C, minimal (night) temperatures were 18, 20 and 22°C, relative humidity was 81, 85 and 88%; the temperature humidity index (THI; see ref. [1] for definition) values at mid-day were 80, 82 and 88.

### Rectal temperature and respiration rate

Respiration rates were determined at 1300 by visually measuring the breathing rates with the aid of a stopwatch. Rectal temperature was measured at 1400 to nearest 0.1°C, with an interchangeable thermistor probe system (model 46TUC Tele-thermometer, Yellow Springs Instruments, Yellow Spring, OH)

### Gross composition, plasmin activity, Na^+ ^and K^+ ^concentrations

Gross composition, the activities of PA, PG, and PL and the concentrations of Na^+ ^and K^+ ^in the milk were determined essentially as described previously [22,27,29].

#### K^+ ^uptake into skim milk vesicles

Milk was fractionated into clear infranatant (Inf; i.e., milk serum devoid of vesicles and casein), skim-milk-derived vesicles (SMV) and casein by a combination of centrifugation and ultracentrifugation [30]. The Inf and SMV were stored at -80°C prior to analysis. The clear Inf was used for measuring K^+ ^channel blocker activity by comparing ^86^Rb uptake into SMV with those obtained after incubation of the SMV in sucrose solution. Protein concentrations in Inf and SMV preparations were assayed by the Bradford method.

The procedure for assaying K^+ ^uptake into the SMV was originally described by Shennan [31] and applied for assessing the activity of the milk-borne potassium channel inhibitor, as described previously [19]; briefly, uptake at 23°C, was initiated by adding 10 μl of ^86^RbCl to a reaction medium consisting of 25 μl of SMV, 325 μl of the working solution (control) or 300 μl of working solution, and 25 μl of test samples. Disposable Pasteur pippets, plugged with polymer filter wool and filled with a cation exchange resin (Dowex, hydrogen form, 50–100 dry mesh, 8% cross-linked), were used to separate intravesicular from extravesicular isotope. Four minutes after the addition of ^86^RbCl, a 200-μl sample of the reaction mixture was applied to the Dowex column, which was then washed with 2 ml of an ice-cold solution that was collected in scintillation vials. The radioactivity of the samples was counted in a Liquid Scintillation Counter (Tri Carb, Hewlett-Packard).

### Preparation of β-CN f (1–28) and measurement of its K^+ ^channel blocking activity

β-CNf (1–28) was selectively precipitated from a plasmin digest of β-CN by using acidified calcium chloride and ethanol [19,32] The peptide was further purified by anion exchange fast protein liquid chromatography and the preparative mode of reversed-phase HPLC, which was also used to collect the peptide [19]. The identity of the major peak collected, which amounted to ~95% of the total peptides, was verified by matrix-assisted laser desorption ionization time-of flight mass spectrometry at the Technion, Haifa, Israel. The K^+ ^uptake into SMV was by comparing SMV derived from the morning milking of control cows and the noon milking of experimental cows, that contained β-CN f (1–28) added at 5 μg ml^-1 ^(~1.5 μM), with a mixture that contained only the incubation solution.

### Statistical analysis

The data were statistically analyzed with the Fit Model procedure for repeated measurements, within the JMP software, Version 5 (SAS Institute, Cary, NC, USA); the between-subject factor was the treatment, and the within-subject factor was time. Differences were considered significant at *P *< 0.05. The model was:



where Y_*ijklm *_= the dependent variable, μ = overall mean, ρ_*i *_= fixed effect of period (pre- or post-treatment; *i *= 1 or 2), α_*j *_= fixed treatment effect *j *(*j *= 1 or 2), C(_*ij*_)*k *= random effect of cow *k *(*k *= 1 to 18) within period *i *and treatment *j*; *γ*_*l *_= effect of day l (l = 1 to 3 or 1 to 9 for milk yield); *αγ*_*il *_= effect of interaction of treatment *j *and day *l*; and *ε*_*ijklm *_= random error associated with cow *k *in period *i *and treatment *j *on day *l*

Differences between treatments for period, or for specific days following treatment were subjected to Student's t-test by means of the Tukey-Kramer Highest Significant Difference (HSD) test.

## Results

Respiration rate per minute measured on the second day of the experiment at 1300 varied significantly among the treatments being 68 ± 7 in treatment C, 102 ± 8 in treatment D and 152 ± 10 in treatment E (*P *< 0.01). Similar ranking (39. 1 ± 1, 39.5 ± 1, 41.6 ± 1, respectively; *P *< 0.01) were recorded with respect to rectal temperatures measured at 1400 on the second day.

**Figure 1:**
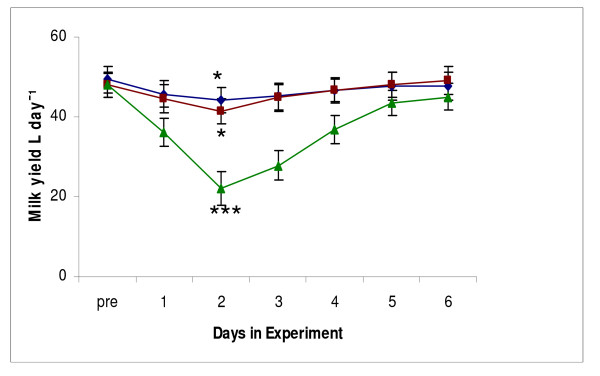
**Effect of treatments on milk yield and its post-treatment recovery**. (diamonds, treatment C; squares, treatment D; triangles, treatment E; * *P *< 0.05 in comparison with pretreatment values; *** *P *< 0.001 in comparison with pretreatment values and treatments C and D).

Milk yields of all cows were in the range 48–49 l/d before the start of the experiment, and did not differ between the treatments at that time (Table 1). All treatments induced a significant drop in milk yield, starting at day 1, which maximized at day 2 of the experiment (Figure 1; Table 1). However, the treatments differed in the extent of the responses: treatment E (a maximal drop of 55.1% in comparison to the pre-treatment yield) > treatment D (-18.2%) > treatment C (-7.9%). After returning the cows to their original group in the afternoon of day 2, treatment's effects on milk yield faded within the next 4 days, the recovery appeared to be slower (within 7 to 10 days) in treatment E (Figure 1).

**Table 1:** Effect of treatments on milk yield and on the diurnal (day vs. night) variations in milk secretion

Treatments	Control (C)	SD	Cooling (D)	SD	Sun (E)	SD
Initial milk yield, Ld^-1^	47.9	3.1	49.3	3.5	48.1	3.6
Night, Lh^-1^	1.80^1^	0.2	1.61^1^	0.2	1.40^1^	0.2
Day, Lh^-1^	1.45^2^	0.3	1.40^2^	0.2	0.65^2^	0.3
Day/Night yield ratio	0.81^a^	0.09	0.88^a^	0.1	0.46^b^	0.07
Total yield, Ld^-1^	44.1	2.5	40.3	2.5	21.1	3.1
Decrease in milk Yield, %	7.9^a^	1.5	18.2^b^	3.6	55.1^c^	5.5

Values marked by different superscript numbers are significantly different within columns; values marked with different superscript letters are significantly different within rows.

Hourly milk secretion rates were determined by dividing the milk recorded at milking by the time interval (~8 h) since the previous milking. Though, within each group, the yield tended to be higher between 0600 and 1400 than between 1400 and 2200, the figures did not differ statistically. Therefore, we pooled the milk yields into night-time yield (2130 to 0530 the next morning) and daytime yield (0530 to 2130 on the same day). Within each group, the milk secretion rate was higher during the night than during the day according to treatments, E difference > treatment D difference = treatment C difference; the daytime yields being lower than the night-time ones by 48, 20 and 19%, respectively. Consequently, the difference in milk yield between treatment E and treatments D and C was smaller during the night than during the day (Table 1).

The treatments did not induce changes in concentrations of lactose, Na (~16 mM), and K (~43 mM), nor,, in the Na/K ratio (~2.7), but elicited increases in total fat and protein concentrations (Table 2). The increases in fat and protein concentration were more apparent in the samples taken at 1400 than in those taken at 0600 am, and the treatments differed in the magnitude of this response, in the order, treatment E > treatment D > treatment C. The increases in fat and protein concentrations elicited by the treatments did not compensate for the drops in their yields caused by the fall in milk yield; however, the differences between night and day secretions of fat and protein were not observed in treatment D (Table 2).

**Table 2:** Effect of treatments on milk composition and on the diurnal (day vs. night) variations in milk composition.

Treatments	Control (C)	SD	Cooling (D)	SD	Sun (E)	SD
Lactose Concentration (%)						
Average	5.10^a^	0.11	5.05^b^	0.10	5.04^b^	0.13
Night	5.10^a^	0.09	5.05^b^	0.11	5.05^b^	0.12
Day	5.09^a^	0.11	5.04^b^	0.09	5.04^b^	0.12
Lactose Yield						
Night, g h^-1^	91.8^1a^	5.5	81.3^1b^	5.4	70.6^1c^	5.6
Day, g h^-1^	73.8^2a^	4.9	70.6^2a^	5.3	35.1^2b^	5.9
Day/Night yield ratio	0.80^a^	0.09	0.87^b^	0.10	0.46^c^	0.08
Decrease in lactose yield, %	7.9^a^	1.5	18.1^b^	3.8	55.0^c^	5.5
Fat concentration (%)						
Average	3.17^a^	0.08	3.25^b^	0.05	3.31^c^	0.09
Night	3.13^a^	0.07	3.05^b^	0.06	3.13^c^	0.07
Day	3.19^a^	0.05	3.35^b^	0.05	3.40^c^	0.09
Fat yield						
Night, g h^-1^	56.3^1a^	3.8	49.1^b^	3.5	43.8^1c^	3.6
Day, g h^-1^	46.3^2a^	3.7	48.6^b^	3.7	22.1^2c^	5.1
Day/Night yield ratio	0.82^a^	0.07	0.99^b^	0.15	0.50^c^	0.12
Decrease in fat yield, %	7.8^a^	1.4	15.9^b^	1.1	49.5^c^	1.2
Protein Concentration (%)						
Average	3.21^a^	0.07	3.31^b^	0.08	3.42^c^	0.07
Night	3.19^a^	0.08	3.17^a^	0.09	3.21^b^	0.07
Day	3.22^a^	0.06	3.38^b^	0.07	3.53^c^	0.08
Protein Yield						
Night, g h^-1^	57.4^1a^	3.9	51.0^1b^	3.1	44.9^1c^	4.5
Day, g h^-1^	46.7^2a^	4.1	47.3^2b^	3.5	22.9^2c^	4.7
Day/Night yield ratio	0.81^a^	0.08	0.93^b^	0.15	51.0^c^	1.0
Decrease in protein yield, %	7.9^a^	1.5	15.8^b^	1.2	49.4^c^	1.8

Values marked with different superscript numbers are significantly different within columns; values marked with different superscript letters are significantly different within rows.

Table 3 shows that acute heat stress led to increased PA and PL activities, and reduction in the PG/PL ratio in the milk; the magnitude of this response diminished in the order treatment E >treatment D > treatment C. As noted above with regard to milk yield and milk composition, there were marked differences between day and night values, and the between-treatments differences in this effect were greater in samples taken at the midday milking and less in those taken at the morning milking (Table 3).

**Table 3:** Effect of treatments on plasminogen activator-plasminogen-plasmin system in milk and on the diurnal (day vs. night) variations in their activity.

Treatments	Control (C)	SD	Cooling (D)	SD	Sun (E)	SD
Average						
Plasminogen activator (PA), unit ml^-1^	11.50^1a^	0.11	15.06^1b^	0.11	17.62^1c^	0.21
Plasminoge (PG), unit ml^-1^	27.51^1a^	0.90	25.60^1b^	0.82	23.91^1c^	0.70
Plasmin (PL),), unit ml^-1^	5.11^1a^	0.11	5.04^1b^	0.09	9.12^1c^	0. 21
Plasminogen/plasmin, ratio	5.38^1a^	0.12	5.08^1b^	0.15	2.62^1c^	0.25
Night						
Plasminogen activator (PA), unit ml^-1^	10.80^2a^	0.30	15.20^2b^	0.15	15.42^2c^	0.19
Plasminoge (PG), unit ml^-1^	30.43^2a^	0.90	23.58^2b^	1.30	29.11^2c^	1.50
Plasmin (PL), unit ml^-1^	5.43^2a^	0.09	4.90^2b^	0.10	6.56^2c^	0.11
Plasminogen/plasmin, ratio	5.60^2a^	0.21	4.81^2b^	0.30	4.44^2c^	0.25
Day						
Plasminogen activator (PA), unit ml^-1^	11.85^3a^	0.17	15.80^3b^	0.15	18.72^3c^	0.29
Plasminoge (PG), unit ml^-1^	26.05^3a^	0.17	26.61^3b^	0.16	21.31^2c^	0.15
Plasmin (PL),), unit ml^-1^	4.95^3a^	0.15	5.11^3b^	0.15	10.40^2c^	0.14
Plasminogen/plasmin, ratio	5.26^3a^	0.16	5.21^3b^	0.17	2.05^2c^	0.25

Values marked with different superscript numbers are significantly different within columns; values marked with different superscript letters are significantly different within rows.

**Figure 2:**
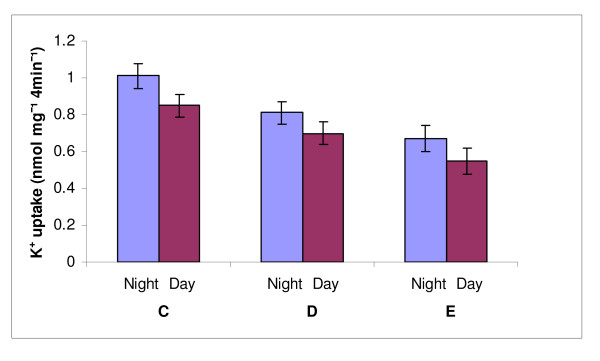
**Effect of treatments and time of sampling on K^+ ^uptake into vesicles derived from milk serum and incubated with the respective infranatant (milk serum devoid of vesicles and casein micelles)**. All the treatments (C, Control, D, treatment D, E, treatment E) and sampling time within treatment (night vs. day) effects were significant at *P *< 0.05).

The data in Figure 2 clearly show that acute heat stress increased the concentration of K^+ ^channel blocker in the milk serum (whey devoid of SMV; Inf) as reflected in the inhibition of K^+ ^uptake into SMV. The magnitude of this response diminished in the order treatment E > treatment D > treatment C.

**Figure 3:**
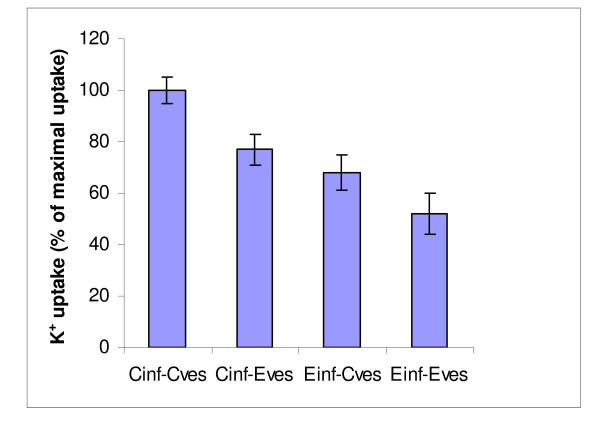
**Effects on K^+ ^uptake into the vesicles, of all combinations of incubating infranatant (milk serum devoid of vesicles and casein micelles) with milk serum-derived vesicles sampled from the control cows at the morning milking and experimental cows at the noon milking**. All combinations differed significantly at *P *< 0.01.

Figure 3 depicts the results of K^+ ^uptake determinations; it presents comparisons of all possible combinations of Inf and SMV sampled from the control cows at the morning milking with those sampled at midday from the experimental cows. The combination of Inf and SMV sampled from the morning milking of the control cows (C_inf_-C_SMV_) served as the reference value. Inhibition of K^+ ^uptake into the vesicles diminished in the order: E_inf_-E_SMV _> E_inf_-C_SMV _> C_inf _- E_SMV_, with values of 52, 68 and 77% of C_inf_-C_SMV_, respectively. The data in Figure 3 are consistent with those in Figure 2, which shows that acute heat stress increased the concentration of K^+ ^channel blocker in the milk serum in proportion to the heat stress level. However, the data also indicate that vesicles (SMV) coming from heat-stressed cows were more responsive to the activity of the K^+ ^channel blocker.

**Figure 4:**
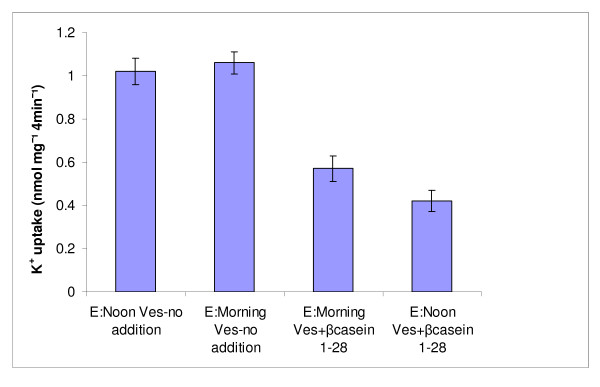
**K^+ ^uptake in milk serum-derived vesicles sampled from morning milking of control cows and noon milking of the experimental cows, with and without the presence 10 μM of β-CN f (1–28)in the incubation medium**. The effect of the presence of β-CN f (1–28) was significant at P < 0.001, whereas the effect of the source of the vesicles was significant at P < 0.01).

Previously, we identified β-CN f (1–28), as the blocker of the putative K^+ ^channel in milk serum-derived vesicles, which serve as model of the K^+ ^channel located on the apical aspect of mammary gland epithelial cells [19]. In order to refine the finding that vesicles derived from heat-stressed cow were more susceptible to the K^+ ^channel blocker than those from unstressed cows, we incubated vesicles derived from the morning milking of control cows and the noon milking of experimental cows, with a fixed amount (~1.5 μM) of βCN f (1–28), and measured their K^+ ^uptakes. The data in Figure 4 clearly show that vesicles derived from the experimental cows were more susceptible to the channel blocking activity of β-CN f (1–28) than those derived from the control cows. In general, the level of inhibition obtained in this experiment was consisted with previous dose-response test results [19].

## Discussion

Our search for the involvement of apically located K^+^- channel in the mechanism of feedback regulation of milk secretion was inspired by the need for extensive apical K^+ ^conductance, since K^+^- concentration of milk is considerably higher than that of plasma [19]. Indeed, K^+ ^channels have been localized in the apical aspect of mammary secretory epithelia [[19,31] and the present study] and have been shown to be associated in milk-born negative feedback regulation of milk secretion [[19], present study]. The voltage-depended nature of the apical K^+^- channel [31], suggests that it belongs to the family of the voltage-gated potassium channels. Indeed, a KCNQ1 (a member of the voltage-gated K^+^- channels) and a number of its accessory subunits were recently localized in the human mammary epithelial cell line, MCF-7 (33). The assembly of KCNQ1 with the accessory units may shift the K^+ ^channel to an open state, associated with hyperpolarization of the membrane potential, which in general facilitates secretary phenomena [33]. Thus, the milk-born regulatory element (i.e., β-CN f (1–28)), which blocks apically located K^+^- channel [[19], present study] should cause depolarization of the membrane potential, and in-turn to down regulate cells metabolism and secretory activity. As discussed below, the present results further support the concept that apically located K^+^- channel and its natural milk-born regulator are involved in negative feedback mechanism that controls milk secretion.

The involvement of PA-PG-PL system in regulation of milk secretion [19,22] and the induction of mammary gland involution [23-26] are well established. The effect of the PA-PG-PL system was related to its contribution to enhanced degradation of the extracellular matrix [20,34]. However, this alternative explanation fell to explain its effect on milk secretion and its sharp increase immediately after the start of active involution [20] (i.e., involution stage I) because in both cases there is no increase in extracellular matrix degradation. The present results as discussed below in line with previous results [22-24], strongly suggest that the PA-PG-PL system works in mammary secretion by increasing casein degradation and liberation of active components, and that β-CN f (1–28) is a principal casein degradation product that involves in negative control of milk secretion.

Animals can adapt to hot environmental conditions by gradual acclimation [8]. As the present experiment was carried out in at mid-summer, it appears logical to assume that the cows were already acclimated to heat stress. Therefore, we first examined whether the treatments induced acute HS responses in the participating cows, and whether the effects of between-treatments differences in the imposed heat loads were significant?

The temperature-humidity index (THI) is a parameter that is widely used to describe the thermal stress imposed by climatic conditions on humans and farm animals. The THI is calculated from a combination of wet- and dry-bulb air temperatures for a particular day: THI values of 70 or less are considered comfortable, 75–78 stressful, and values greater than 78 cause extreme distress [2,6]. According to these criteria the cows in the three treatments were under extreme heat stress during all the days of the experiment, though the HS condition worsened quite dramatically on the second day. Nevertheless, the THI in this case obviously could not account for the differences between treatments in milk yield, in light of previous experimental findings that the THI accounted for only small proportion of the variance of heat stress-related milk yield in dairy cows [1,35]. This is in part because of wide variations between individuals, in part because the animal is related to its environment in a much more complex manner than is represented by this index, and in part because of the cooling measures applied, that help to relieve some of the heat stress. A major factor that is not taken into account in the THI is solar radiation [1]. Multiple regression analysis that used respiration rate and body temperature as the dependent response variables to derive an index of effective temperature load has shown that the constant associated with radiation (black globe temperature) was three times as great as that associated with ambient temperature (dry-bulb temperature) [36].

In order to solve the above problems, thermal physiologists adopted the stress-strain relationship from physical sciences as an applicable concept, with strain referring to an internal displacement from the resting or basal state brought about by external stress [1,37]. Based on the differences in respiration rate and rectal temperature, the heat-load-related strain response diminished in the order, treatment E > treatment D > treatment C. A more detailed description than that provided by the classical thermoneutral zone concept of the relation between an animal and its environment was proposed [1], according to which, treatment E brought the cows deep into the noxious stage, in which coping attempts to maintain the correct body temperature are markedly unsuccessful, and drove them too close to the extreme stage, which is defined as that in which a vicious cycle of rising body temperature starts that eventually may become lethal. Therefore, the experiment was terminated at this stage, and the cows were returned to their normal, most optimal conditions (with access to shade and cooling). This situation is consistent with previous reports that if hot conditions were sudden and prolonged, as is often the case in the Mediterranean areas and in some regions of the United States, cows cannot be regarded as acclimatized, as such heat-stress conditions are marked by falls in milk production and feed intake, and increased body temperature [1,38].

Lactation is a physiological process that presents a substantial challenge to the homeostasis of the cardiovascular and fluid secretory system [1,10,12,13]. The acute and large decrease in milk secretion, particularly in treatment E, may, therefore, be considered as having vital importance that makes it necessary to enable the cows to survive the HS. Thus, our present results conflict with the concept that the initial reactions of the animal to acute heat stress represent merely an emotional rather than a thermoregulatory response (see discussion in ref. [1]).

During the summer months in Israel and hotter parts of the USA, milk yields, as well as milk protein fat contents, was found to be reduced, even in dairy herds in which the cows were cooled, which indicates that these reductions form part of their adaptive response [2,6,38]. On the other hand, acute stress in response to intramammary treatment with dexamethasone or to dehydration resulted in more intense inhibition of lactose and fluid secretion than of fat and protein secretion, which was reflected in increased fat and protein concentrations in milk, though these increases did not compensate for the overall reduction in their yields [19,30]. Thus, the present results regarding the effect of HS on fat and protein concentrations are consistent with the above-noted response to acute stress. In keeping with previous findings [19,20], in this paper we present evidence that the acute reduction in milk secretion under heat stress is associated with activation of the PA-PG-PL-release of β-CN (1–28) system. It has been shown that decrease in Na concentration, increase in K concentration and the consequent decrease in the Na/K ratio is a sensitive indication of the disruption of the tight junction of the mammary gland epithelial cells, which relates to differing ion contents in milk and blood plasma [22,39]. In previous studies stress-induced changes in milk secretion and composition were detected without evidence for disruption of the tight junction of mammary gland epithelial cells [19,22,27], which is also consistent with the present findings.

Isolation and regrouping has been shown to impose an emotional stress that is associated with reduced milk yield and activation of the systemic stress response [39,40]. Thus, the significant reduction in milk yield in treatment C, may also relate to activation of a local negative-feedback control, in response to emotional stress. However, we could not test this hypothesis because of the confounding effect of the increase in HS between the start and day 2 of the experiment, which independently could have activated the negative-feedback regulatory system.

The present study highlighted two previously unreported features of this local negative-feedback regulatory system.

First, that is a very fast-responding and flexible system, which can respond to diurnal (night vs. day) changes in the ambient conditions. Such a precise level of response to fluctuating conditions may be made possible if the mammary gland cells themselves serve as a regulatory afferent component in the regulatory system. In keeping with such a hypothesis, there is evidence that local heat shock induces endogenous hyperfibrinolysis, which is equivalent to enhanced caseinolysis in the present case, by upregulation of plasminogen activators [41].

The hypothesis that HS induces a short-term rapid regulatory response is consistent with evidence that in lactating cows under commercial production conditions, the effects of heat stress that may be experienced under exposure to high ambient temperatures during the day appears to be ameliorated when temperatures fall at night, and that lack of a cool night-time ambient temperature intensifies the reduction in milk yield [1,38].

Secondly, we demonstrated that expression of potassium channels on the apical membrane serves as a regulatory component in the negative-feedback system, by increasing the apical-membrane K channel sensitivity to β-CN f (1–28) levels, and this, most likely, further contributes to the accuracy and efficacy of the system. A turnover with biological half-life of 7.3 h was demonstrated for a K_ATP _channel [42]; thus, the rapid night to day shift in the sensitivity to K^+ ^uptake through apical-membrane-derived vesicles is theoretically possible.

## Conclusion

We presented evidence that the acute phase in the regulatory inhibition of milk secretion in cows subjected to heat stress is related to upregulation of the local PA-PG-PL-β-CN f (1–28) peptide in milk and that this peptide in turn down regulate the activity of K^+ ^channels on apical-membranes derived vesicles. Further research is needed, to determine the nature of the interaction of β-CN f (1–28) with regulatory elements in the apical membrane of mammary gland epithelial cells, and to identify these channels and the components of the inward signal transduction. We are currently pursuing the hypothesize that the putative apical K^+ ^channels belong to the family of voltage-gated channels and that β-CN f (1–28) causes membrane depolarization, explaining its milk down-regulatory effect.

## Competing interests

The authors declare that they have no competing interests.

## Authors' contributions

NS conceived the project, supervised all of its stages, and wrote the manuscript; FS carried out the biochemical analysis; DS carried out the potassium uptake measurements. All authors read and approved the final manuscript.
